# Supplementary material for: Multi-angle pulse shape detection of scattered light in flow cytometry for label-free cell cycle classification
Source: Commun Biol. 2021 Sep 30;4:1144. doi: 10.1038/s42003-021-02664-3 (PMC8484341; doi:10.1038/s42003-021-02664-3)
Supplement: Supplementary file 2 — Supplementary Information [file 42003_2021_2664_MOESM2_ESM.pdf]

Supporting Information

Multi-angle pulse shape detection of scattered light in flow cytometry for label-free cell cycle classification

Daniel Kage[1], Kerstin Heinrich[1], Konrad v. Volkmann[2], Jenny Kirsch[1], Kristen Feher[3], Claudia Giesecke-Thiel[4,*], Toralf Kaiser[1,*]

[1] *German Rheumatism Research Centre Berlin (DRFZ) - Flow Cytometry Core Facility, Charitéplatz 1 (Virchowweg 12), 10117 Berlin, Germany*

[2] *APE Angewandte Physik und Elektronik GmbH, Plauener Straße 163-165 / Haus N, 13053 Berlin, Germany*

[3] *EMBL Australia Node in Single Molecule Science, School of Medical Sciences, University of New South Wales, Sydney, Australia*

[4] *Flow Cytometry Facility, Max Planck Institute for Molecular Genetics, Ihnestraße 63-73, 14195 Berlin, Germany & formerly at the German Rheumatism Research Centre Berlin (DRFZ) – Cell Biology*

*equal contribution

Corresponding authors:

Toralf Kaiser, kaiser@drfz.de,
Deutsches Rheuma-Forschungszentrum (DRFZ) Berlin
Charitéplatz 1
10117 Berlin

Claudia Giesecke-Thiel, giesecke@molgen.mpg.de
Max Planck Institute for Molecular Genetics
Ihnestraße 63-73
14195 Berlin

1. Single-cell gating

Gating for single cells was applied before MAPS-FC analysis. The gating strategy is displayed in Figure S1.

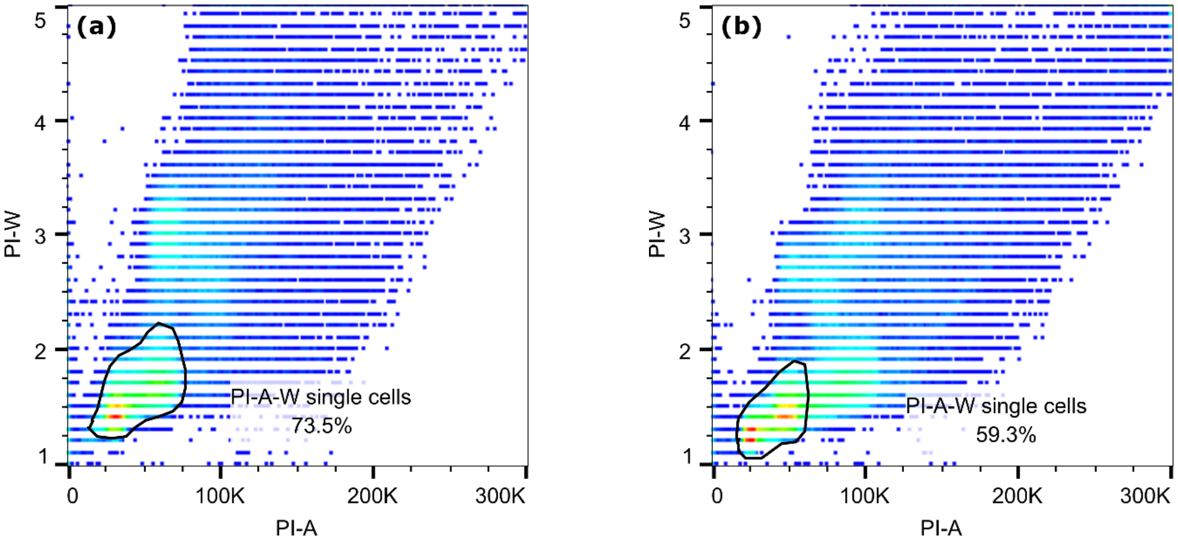

Figure S1: Gating for single cells based on PI-A and PI-W parameters in (a) HEK293 cells and (b) Jurkat cells.

2. Metric for assignment of clusters to the cell cycle phases in HEK cells

The specificity of the clusters resulting from the pulse shapes with respect to staining was evaluated as follows: for each cluster, the fraction of cells in the cell cycle phases G1, S, and G2/M (as defined by the staining) was determined. These fractions were compared with the sample average. We define the enrichment $x(P,c)$ of a cluster c with cells from cell cycle phase P as

$$x(P,c) = \frac{\left(\frac{N(P,c)}{N(\cdot,c)}\right)}{\left(\frac{N(P,\cdot)}{N(\cdot,\cdot)}\right)} \quad (1)$$

where $N(P,c)$ is the absolute number of cells from cluster c that fall into the gate of cell cycle phase P. The cell cycle phase P is one of the phases G1, S, and G2/M. The cluster c denotes one of the 64 combined clusters. The symbol $\cdot$ is a placeholder for all phases or clusters (e.g., $N(\cdot,\cdot)$ means all single cells of the whole sample). Thus, for each cluster there are three values of x, one for each cell cycle phase.

If $x(P,c) = 1$, the respective cluster c contains the same fraction of cells in phase P as the overall sample. In the case that $x(P,c) > 1$, the cluster c contains a larger fraction of cells in phase P than the overall sample which means that the cluster is enriched with cells from this phase. For $x(P,c) < 1$, the cluster conatins a smaller fraction of cells in phase P than the overall sample which means that the cluster is depleted of cells from this phase. Consequently, clusters that are specific for a certain cell cycle phase must preferentially show a high enrichment for this phase and a strong depletion for the other two phases.

Using these enrichment values for all clusters in all three phases, we set up boundaries for classification of a cluster. The enrichment values are sorted by size for each cluster: $x(P_{\mathrm{max}},c) > x(P_{\mathrm{mid}},c) > x(P_{min},c)$. For each cluster, the following conditions are checked:

- if $x(P,c) > 1$ for only one phase, and $x(P_{\mathrm{max}},c) > 4 \cdot x(P_{\mathrm{mid}},c)$, the cluster is assigned to this phase with largest enrichment
- if $x(P,c) > 1$ for one or more phases, and $x(P_{\mathrm{min}},c) < 0.5$, the cluster is assigned to the two phases with the larger values of x
- if $x(P_{\mathrm{min}},c) < 0.5$ and $x(P_{\mathrm{mid}},c) > 0.75$, the cluster is assigned to the two phases with the largest enrichment
- if none of the above conditions is fulfilled, the cluster is considered unspecific

Only clusters above a minimum size of 250 cells were considered. Applying the rules listed above, the clusters can be classified as shown in Figure S2(a). Each black circle represents one of the 8x8=64 combined clusters. The area of the circles is

proportional to the relative size of the cluster. The colored boxes show the assignment of the clusters to the cell cycle phases defined by the staining. For the phases G1 and G2/M, clusters were found that could be exclusively assigned to these phases. Most clusters cover two neighboring cell cycle phases. This is in agreement with the fluorescence intensity distributions shown in the main manuscript. The assignment of the clusters to the cell cycle phases confirms that the FSCL channel provides a coarse distinction between G1 and G2/M cells and the FSCU channel adds information to distinguish between neighboring phases. In Figure S2(b), the events from all clusters in each group are shown in an overlay FSCM-SSC dotplot. Obviously, a discrimination in the conventional dotplot would not have been possible. The fluorescence intensity distribution for each group of clusters is given in Figure S2(c).

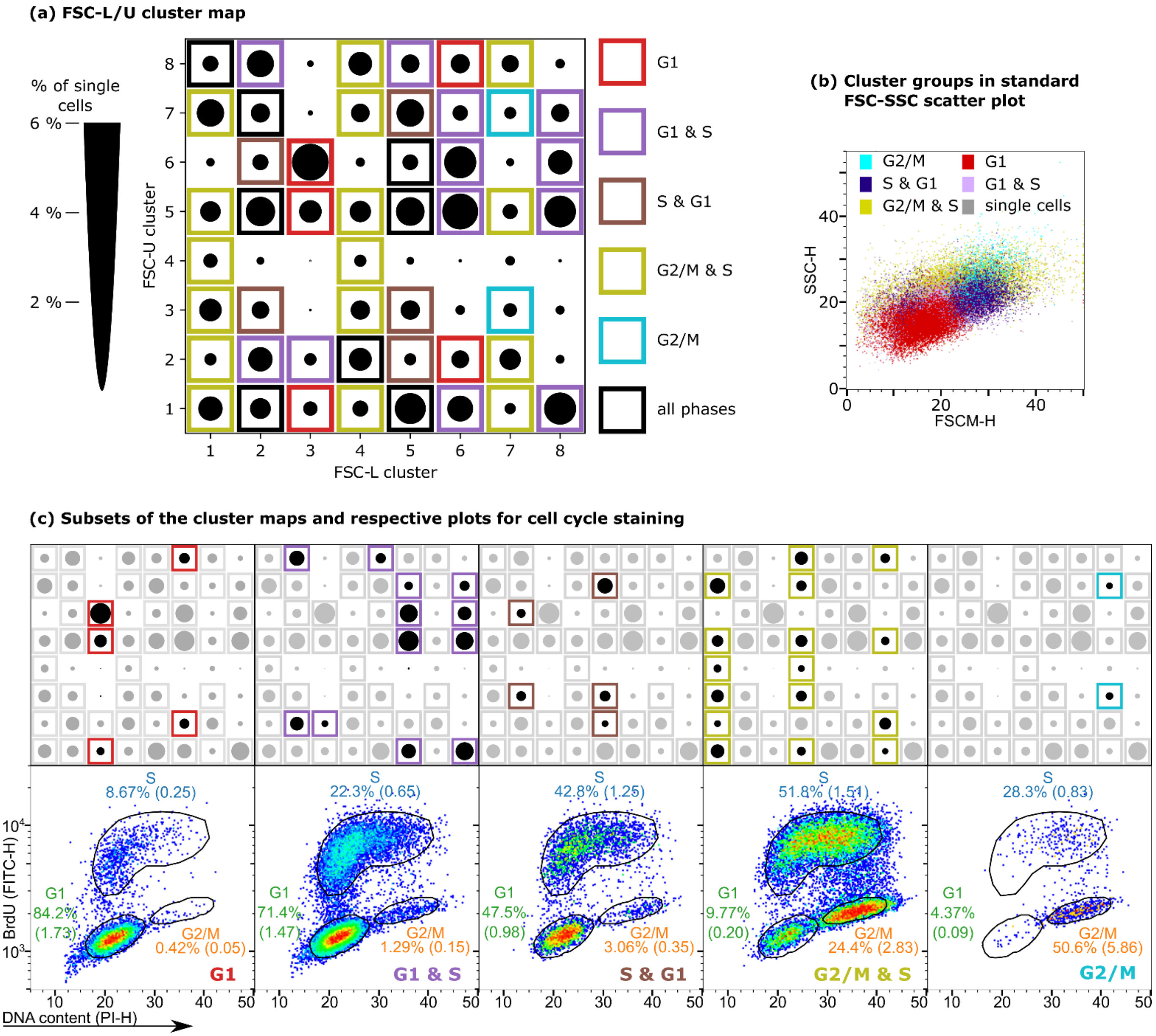

Figure S2: Classification of the clusters into groups according to the cell cycle staining. (a) Assignment of the clusters to the cell cycle phases based on the enrichment, Eq. (1). The area of the circles is proportional to the relative size of the clusters. (b) Group-wise FSCM-SSC overlay dotplot. (c) Fluorescence intensity distribution of the events from all clusters in each group. The percentages give the fraction of cells within the respective gate. The numbers in parentheses show the enrichment.

3. Cell cycle analysis of Jurkat cells by MAPS-FC

In addition to the experiments on the HEK293 cells presented in the main manuscript, MAPS-FC was also tested with Jurkat cells.

3.1. Cell cycle characterization

Cells of the Jurkat cell line were prepared according to the description in the Methods Section of the main manuscript. A total of 10^5 events was acquired with the MAPS-FC setup. Single cell gating was performed based on fluorescence area and width parameters of PI, cf. Figure S1(b). The events within this gate were analyzed for their cell cycle phases (G1, S, G2/M) according to the DNA (PI) and BrdU (FITC) staining, as shown in Figure S3(a). The events within these populations are displayed in the standard FSCM-SSC scatter overlay plot in Figure S3(b). The overlay shows that the FSCM signals increase with increasing cell division, indicating cell growth.

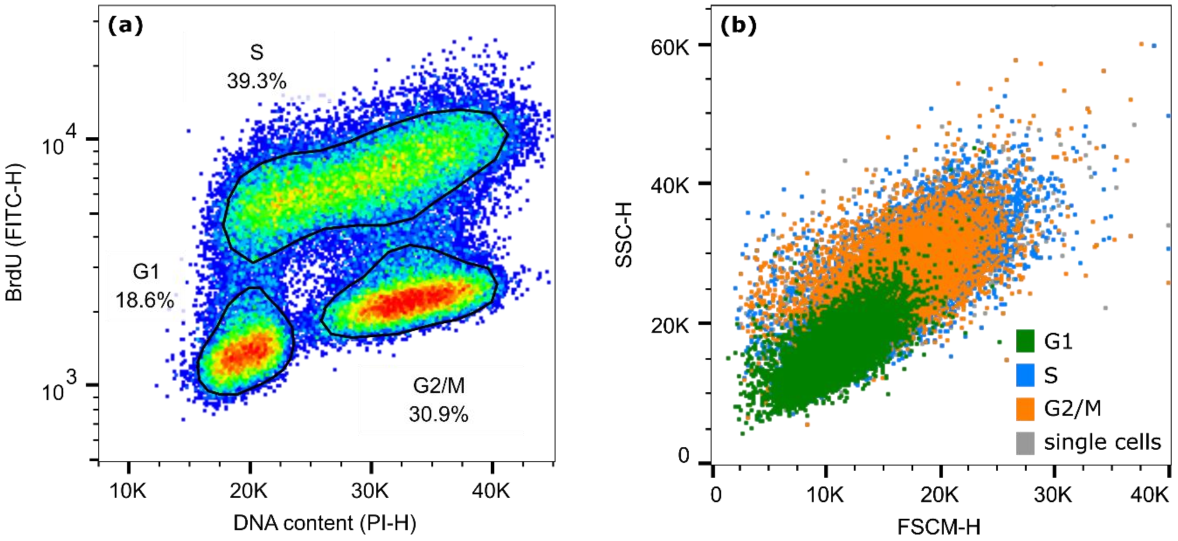

Figure S3: Common cell cycle analysis of stained Jurkat cells, gated for single-cell events before. (a) Fluorescence intensity pseudocolor plot for PI and BrdU-FITC staining. The populations are assigned to the phases of the cell cycle as indicated. (b) Location of the cell cycle populations in a standard FSCM-SSC dotplot.

Pulse shapes from the respective populations in Figure S3(a) are displayed in Figure S4 for the SSC channel and two FSC channels, FSCL and FSCU (definitions see Methods Section on the optical setup of the main manuscript). The SSC pulse shapes (first column Figure S4) do exhibit some broadening of the pulse across the cell cycle phases. However, the general shape of the pulses stays unaltered. In FSCL (second column Figure S4), one can observe that the position and width of the first peak is virtually unaffected by the cell cycle phase. The following dip, however, slightly shifts to later time points from G1 over S to G2/M. A shift of the second peak is present between G1 and G2/M while cells in the S phase present a larger variation of the position of this second peak. The signals in the FSCU channel (third column Figure S4) exhibit generally more variation of the pulse shapes within the cell cycle phases. While the first peak again seems to be unaffected, the dip broadens with progression through the cell cycle from G1 over S to G2/M. Moreover, the position of the second peak again significantly shifts in time across the cell cycle phases. The third FSC channel, FSCM, did not provide significant information beyond the other two in the case of Jurkat cells.

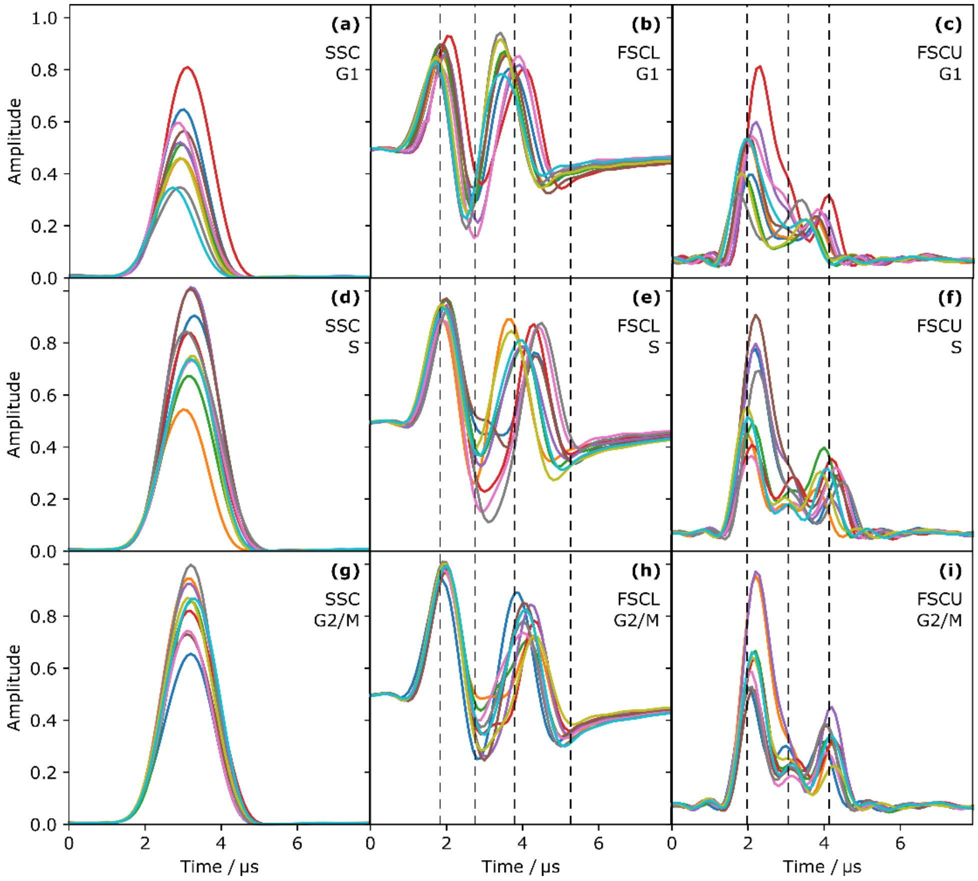

Figure S4: Exemplary pulse shapes for SSC, FSCL, and FSCU for the three cell cycle phases G1, S, and G2/M of Jurkat cells. Each row corresponds to one phase of the cell cycle. Each column corresponds to one detector: SSC (a, d, g), FSCL (b, e, h), FSCU (c, f, i), G1 (a-c), S (d-f), G2/M (g-i). The vertical lines serve as guides to the eye for comparison of the pulse shapes.

For the feature extraction from the pulse shapes, the Haar wavelet at feature level 4 was used. Subsequently, *k*-means clustering was performed and thereby the events were assigned to 8 clusters in each channel. The clusters in the FSCL and FSCU channels were combined in conjunction to give 8x8=64 combined clusters. Figure S5(a) shows plots of the fluorescence intensity distribution of each combined cluster which provides an overview over the composition of all clusters in terms of the fluorescent cell cycle staining. The fluorescence intensity distributions for five exemplarily chosen clusters are given in Figure S5(b) along with additional details about the fractions of cells in the three cell cycle phases and the enrichment of cluster with cells from the respective phases.

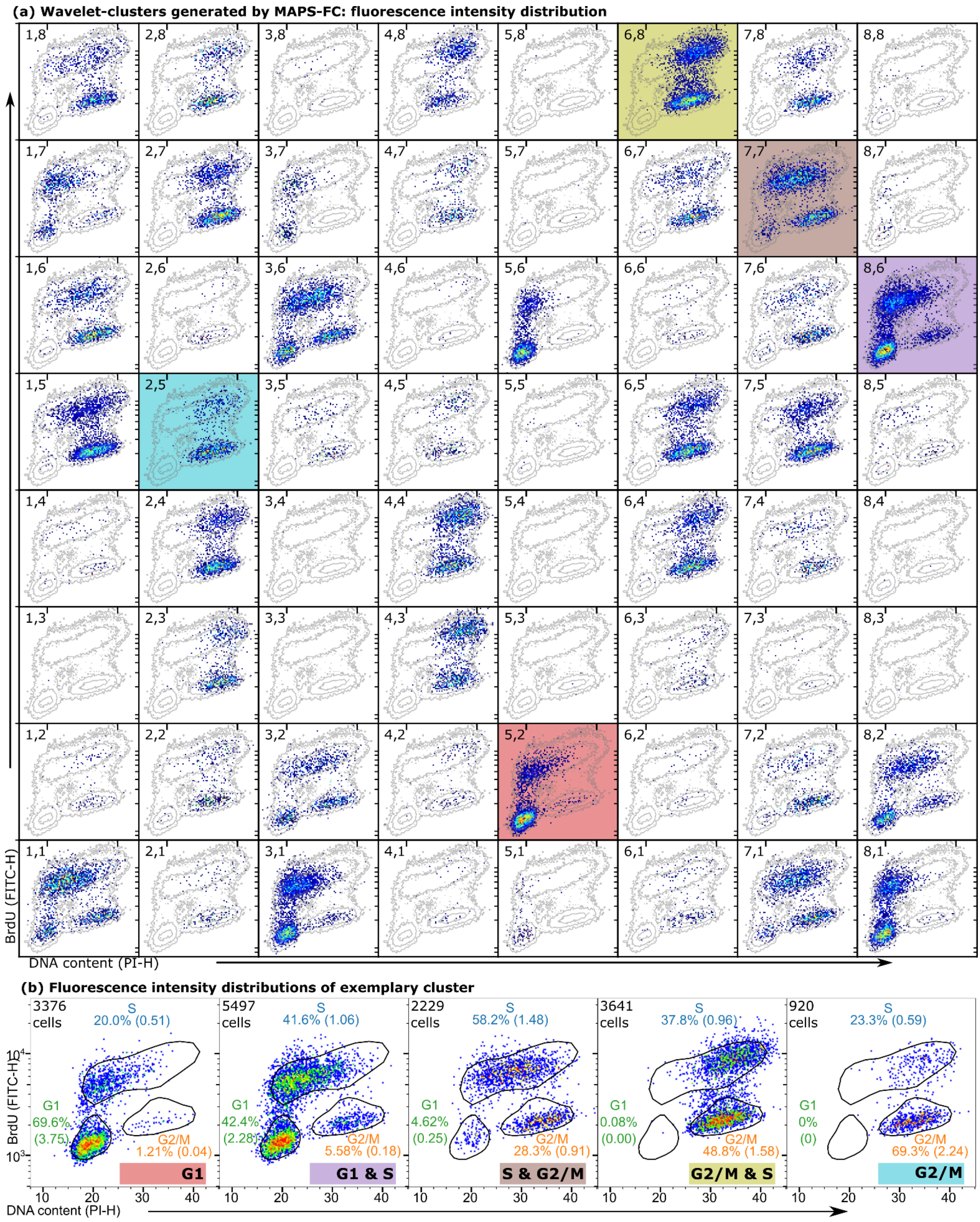

Figure S5: Cell cycle analysis with MAPS-FC. (a) Fluorescence intensity distribution in each cluster (8 clusters/channel, 64 combined clusters). Contour lines indicate the distribution in the overall sample. (b) Fluorescence intensity pseudocolor plots of exemplary clusters chosen from panel (a) as indicated. Additionally, the fractions of events in the cell cycle gates (defined by the staining) are given. Numbers in parenthesis are enrichment values, cf. Eq. (1).

As described in the preceding Section for the HEK cells, the clusters were classified based on their enrichment with cells of a certain phase. The boundaries were slightly adapted to the situation for this sample. Here, for classification as purely one phase, the maximum enrichment needs to be 3 times the next largest enrichment instead of 4 times but the principle stays the same. The assignment of the clusters to the cell cycle phases is shown in Figure S6(a). Figure S6(b) shows the events from all cluster in each group in the conventional FSCM-SSC dotplot. The fluorescence intensity distribution within the cluster groups is displayed in Figure S6(c).

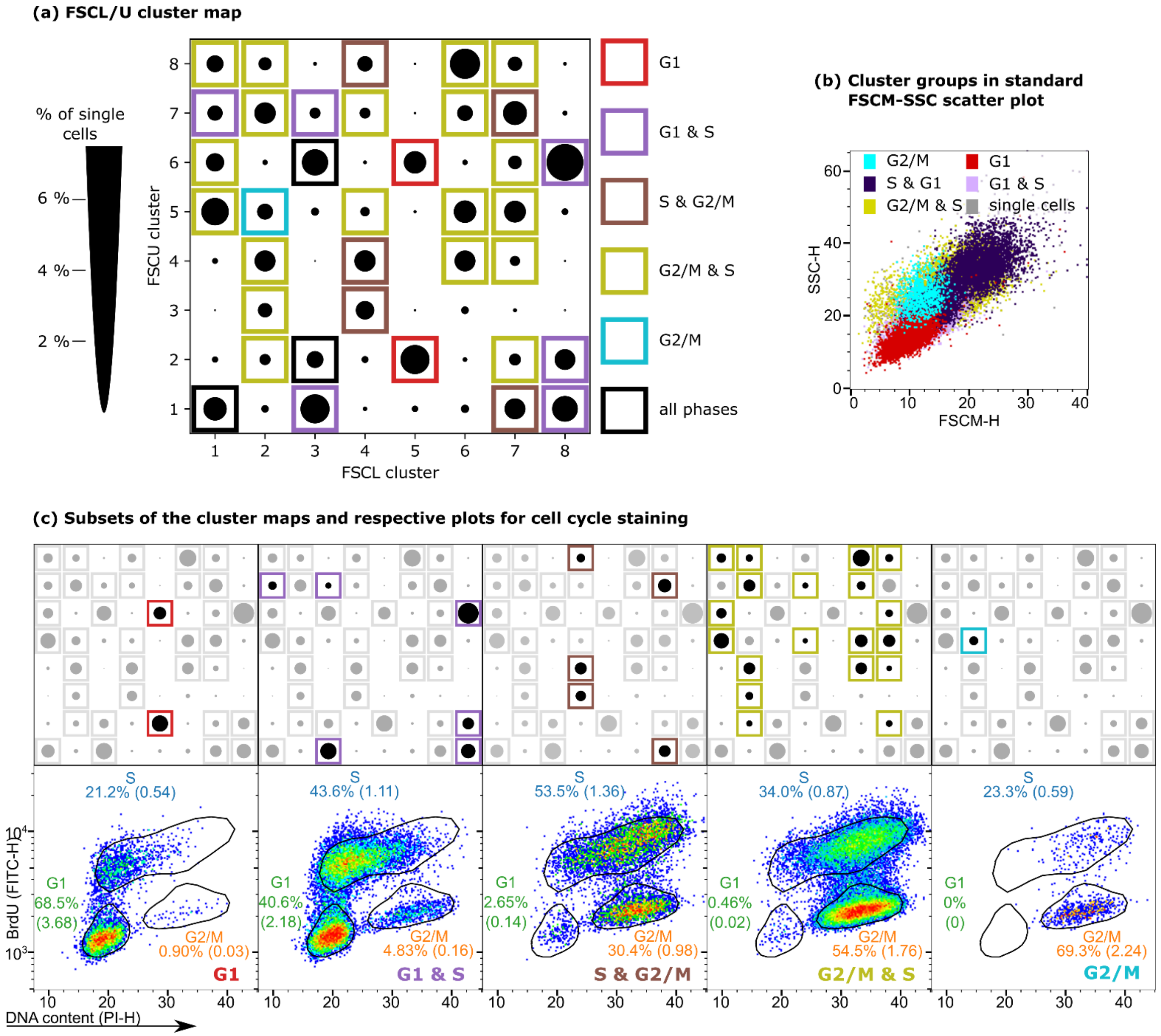

Figure S6: Classification of the clusters into groups according to the cell cycle staining. (a) Assignment of the clusters to the cell cycle phases based on the enrichment, Eq. (1). The area of the circles is proportional to the relative size of the clusters. (b) Group-wise FSCM-SSC overlay dotplot. (c) Fluorescence intensity distribution of the events from all clusters in each group. The percentages give the fraction of cells within the respective gate. The numbers in parentheses show the enrichment.

3.2. Conclusion

The pulse shapes and differences between the pulse shapes across the cell cycle phases in Jurkat cells were similar to those found in HEK293 cells presented in the main manuscript. The MAPS-FC method classified the events into cluster groups that could be assigned to individual cell cycle phases or two neighboring phases as defined by the fluorescent staining. This is in line with the results on the HEK293 cells presented in the main manuscript. Overall, the experiments on this second cell line support the findings presented in the main manuscript and therefore underline the feasibility of MAPS-FC and wavelet-cluster method for cell cycle analysis.
