# Supplementary material for: Multi-angle pulse shape detection of scattered light in flow cytometry for label-free cell cycle classification
Source: Commun Biol. 2021 Sep 30;4:1144. doi: 10.1038/s42003-021-02664-3 (PMC8484341; doi:10.1038/s42003-021-02664-3)
Supplement: Supplementary file 3 — Reporting Summary [file 42003_2021_2664_MOESM3_ESM.pdf]

nature research

Corresponding author(s): Toralf Kaiser

Last updated by author(s): Sep 6, 2021

Reporting Summary

Nature Research wishes to improve the reproducibility of the work that we publish. This form provides structure for consistency and transparency in reporting. For further information on Nature Research policies, see our Editorial Policies and the Editorial Policy Checklist.

Statistics

For all statistical analyses, confirm that the following items are present in the figure legend, table legend, main text, or Methods section.

n/a	Confirmed	
☒	☐	The exact sample size (*n*) for each experimental group/condition, given as a discrete number and unit of measurement
☒	☐	A statement on whether measurements were taken from distinct samples or whether the same sample was measured repeatedly
☒	☐	The statistical test(s) used AND whether they are one- or two-sided<br>*Only common tests should be described solely by name; describe more complex techniques in the Methods section.*
☒	☐	A description of all covariates tested
☒	☐	A description of any assumptions or corrections, such as tests of normality and adjustment for multiple comparisons
☐	☒	A full description of the statistical parameters including central tendency (e.g. means) or other basic estimates (e.g. regression coefficient) AND variation (e.g. standard deviation) or associated estimates of uncertainty (e.g. confidence intervals)
☒	☐	For null hypothesis testing, the test statistic (e.g. *F*, *t*, *r*) with confidence intervals, effect sizes, degrees of freedom and *P* value noted<br>*Give P values as exact values whenever suitable.*
☒	☐	For Bayesian analysis, information on the choice of priors and Markov chain Monte Carlo settings
☒	☐	For hierarchical and complex designs, identification of the appropriate level for tests and full reporting of outcomes
☒	☐	Estimates of effect sizes (e.g. Cohen's *d*, Pearson's *r*), indicating how they were calculated

Our web collection on statistics for biologists contains articles on many of the points above.

Software and code

Policy information about availability of computer code

Data collection	custom-made flow cytometry data acquisition software, INSPIRE (Version 200.1.388.0) for Amnis ImageStream X MkII (Luminex)
Data analysis	FlowJo 10.6.2 (BD), IDEAS 6.2 (Luminex), Matlab R2019b (The MathWorks), Python, Microsoft Excel

For manuscripts utilizing custom algorithms or software that are central to the research but not yet described in published literature, software must be made available to editors and reviewers. We strongly encourage code deposition in a community repository (e.g. GitHub). See the Nature Research guidelines for submitting code & software for further information.

Data

Policy information about availability of data

All manuscripts must include a data availability statement. This statement should provide the following information, where applicable:

- Accession codes, unique identifiers, or web links for publicly available datasets
- A list of figures that have associated raw data
- A description of any restrictions on data availability

The datasets generated and analyzed during the current study are available from an open repository (DOI: 10.5281/zenodo.5235706). Any remaining information can be obtained from the corresponding author upon reasonable request.

Field-specific reporting

Life sciences study design

All studies must disclose on these points even when the disclosure is negative.

Sample size	Cells were seeded in defined density one day before treatment. Cells were counted using a Neubauer counting chamber. At flow cytometer defined number of single-cell events were used for analysis.
Data exclusions	Only events from single cells according to common single-cell gating were included in further analysis.
Replication	The method was tested with two different cell lines and with multiple separately prepared cell samples. Some experiments failed due to sample preparation issues. All other experiments generally confirmed the feasibility of the approach.
Randomization	Randomization within cell samples is intrinsic.
Blinding	Blinding was not necessary/possible.

Reporting for specific materials, systems and methods

We require information from authors about some types of materials, experimental systems and methods used in many studies. Here, indicate whether each material, system or method listed is relevant to your study. If you are not sure if a list item applies to your research, read the appropriate section before selecting a response.

Materials & experimental systems

n/a	Involved in the study	
☐	☒	Antibodies
☐	☒	Eukaryotic cell lines
☐	☐	Palaeontology and archaeology
☐	☐	Animals and other organisms
☐	☐	Human research participants
☐	☐	Clinical data
☐	☐	Dual use research of concern

Methods

n/a	Involved in the study	
☐	☐	ChIP-seq
☐	☒	Flow cytometry
☐	☐	MRI-based neuroimaging

Antibodies

Antibodies used	BrdU Monoclonal Antibody, FITC, Bu20a, Thermo Fisher Scientific, Cat.No. 11-5071-42, Lot. No. 1982681, 5 µl for 1x10e6 cells
Validation	Using a control sample of cells which were not treated with BrdU. A staining of these cells using the BrdU-FITC antibody showed no positive BrdU specific signal.

Eukaryotic cell lines

Policy information about cell lines

Cell line source(s)	All cell lines were originally obtained from DSMZ.
Authentication	None of cell lines used were authenticated.
Mycoplasma contamination	Cell lines were not tested for mycoplasma contamination.
Commonly misidentified lines (See ICLAC register)	N/A

Palaeontology and Archaeology

Specimen provenance	N/A
Specimen deposition	N/A
Dating methods	N/A

☐ Tick this box to confirm that the raw and calibrated dates are available in the paper or in Supplementary Information.

Ethics oversight	N/A

Note that full information on the approval of the study protocol must also be provided in the manuscript.

Animals and other organisms

Policy information about studies involving animals; ARRIVE guidelines recommended for reporting animal research

Laboratory animals	N/A
Wild animals	N/A
Field-collected samples	N/A
Ethics oversight	N/A

Note that full information on the approval of the study protocol must also be provided in the manuscript.

Human research participants

Policy information about studies involving human research participants

Population characteristics	N/A
Recruitment	N/A
Ethics oversight	N/A

Note that full information on the approval of the study protocol must also be provided in the manuscript.

Clinical data

Policy information about clinical studies

All manuscripts should comply with the ICMJEguidelines for publication of clinical research and a completedCONSORT checklist must be included with all submissions.

Clinical trial registration	N/A
Study protocol	N/A
Data collection	N/A
Outcomes	N/A

Dual use research of concern

Policy information about dual use research of concern

Hazards

Could the accidental, deliberate or reckless misuse of agents or technologies generated in the work, or the application of information presented in the manuscript, pose a threat to:

No	Yes	
☒	☐	Public health
☒	☐	National security
☒	☐	Crops and/or livestock
☒	☐	Ecosystems
☒	☐	Any other significant area

Experiments of concern

Does the work involve any of these experiments of concern:

No	Yes	
☒	☐	Demonstrate how to render a vaccine ineffective
☒	☐	Confer resistance to therapeutically useful antibiotics or antiviral agents
☒	☐	Enhance the virulence of a pathogen or render a nonpathogen virulent
☒	☐	Increase transmissibility of a pathogen
☒	☐	Alter the host range of a pathogen
☒	☐	Enable evasion of diagnostic/detection modalities
☒	☐	Enable the weaponization of a biological agent or toxin
☒	☐	Any other potentially harmful combination of experiments and agents

ChIP-seq

Data deposition

☐ Confirm that both raw and final processed data have been deposited in a public database such as GEO.

☐ Confirm that you have deposited or provided access to graph files (e.g. BED files) for the called peaks.

Data access links<br>*May remain private before publication.*	N/A
Files in database submission	N/A
Genome browser session<br>(e.g. UCSC)	N/A

Methodology

Replicates	N/A
Sequencing depth	N/A
Antibodies	N/A
Peak calling parameters	N/A
Data quality	N/A
Software	N/A

Flow Cytometry

Plots

Confirm that:

☒ The axis labels state the marker and fluorochrome used (e.g. CD4-FITC).

☒ The axis scales are clearly visible. Include numbers along axes only for bottom left plot of group (a 'group' is an analysis of identical markers).

☐ All plots are contour plots with outliers or pseudocolor plots.

☒ A numerical value for number of cells or percentage (with statistics) is provided.

Methodology

Sample preparation	Cells were seeded at 20 -30 % confluence and were cultivated for 20 h. Then the cells were treated with 60 µM BrdU for 1 h. Cells were washed twice with 1x PBS (Th. Geyer) and harvested for fixation. Adherent cells (HEK293) were detached by using Trypsin/EDTA. Cells were spin down and resuspended in 500 µl PBS. 4.5 mL of cold methanol/acetone (-20 °C) were added to 1x10e7 cell suspension in a drop wise manner while vortexing. Cells were incubated overnight at -20 °C. After fixation, cells were centrifuged and washed once in PBS. Cells were then treated first with 2 M HCl/0.5 % Triton X-100 for 30 min at room temperature. After centrifugation, cells were neutralized by resuspending in Na2B4O7 and incubation for 2 min at RT. Cells were washed once in PBS/1 % BSA and re-suspended in PBS/1 % BSA/0.5 % TWEEN20 and incubated with anti-BrdU-FITC antibody for 1 hour. After washing the cells using PBS/0,5% BSA, cells were incubated with Propidium Iodide (PI) staining solution (PBS containing 50 µg/mL PI, 100 µg/mL RNase A, 2 mM MgCl2) for 20 min at RT.

	For cell cycle arrest, cells were seeded at 20 -30 % confluence and treated with 10 μM RO-3306 for 20 h. Cells were released from G2 arrest by washing twice with fresh medium for 3 min. Analysis of cell cycle stage was performed between 0 h and 5 h after substitution of RO-3306 with fresh media. Before the cells were harvested, they were treated with 60 μM BrdU for 1h. Further sample preparation was as described before.
Instrument	modified LSR II (BD), FACSAria II (BD), Amnis ImageStream X MkII (Luminex)
Software	INSPIRE Version 200.1.388.0 and IDEAS 6.2 (Luminex), FlowJo 10.6.2 (BD), Matlab R2019b (The MathWorks), Python, Microsoft Excel
Cell population abundance	N/A
Gating strategy	single cell gating was performed based on PI-area and PI-width signals, cell cycle phases are determined by three populations in a FITC-PI pseudocolor plot

☒ Tick this box to confirm that a figure exemplifying the gating strategy is provided in the Supplementary Information.

Magnetic resonance imaging

Experimental design

Design type	N/A
Design specifications	N/A
Behavioral performance measures	N/A

Acquisition

Imaging type(s)	N/A
Field strength	N/A
Sequence & imaging parameters	N/A
Area of acquisition	N/A

Diffusion MRI ☐ Used ☐ Not used

Preprocessing

Preprocessing software	N/A
Normalization	N/A
Normalization template	N/A
Noise and artifact removal	N/A
Volume censoring	N/A

Statistical modeling & inference

Model type and settings	N/A
Effect(s) tested	N/A

Specify type of analysis: ☐ Whole brain ☐ ROI-based ☐ Both

Statistic type for inference (See Eklund et al. 2016)	N/A
Correction	N/A

Models & analysis

n/a	Involved in the study
☒	☐ Functional and/or effective connectivity
☒	☐ Graph analysis
☒	☐ Multivariate modeling or predictive analysis
